# Supplementary material for: Hierarchical mechanisms control the clearance of DNA lesion–stalled RNA polymerase II
Source: Nat Commun. 2026 Jan 19;17:1647. doi: 10.1038/s41467-026-68413-4 (PMC12904859; doi:10.1038/s41467-026-68413-4)
Supplement: Supplementary file 1 — Supplementary Information [file 41467_2026_68413_MOESM1_ESM.pdf]

Supplementary Information for

Hierarchical mechanisms control the clearance of DNA lesion–stalled RNA polymerase II

Paula J. van der Meer[1#], George Yakoub[1#], Kotaro Tsukada[2], Yuka Nakazawa[3], Tomoo Ogi[2,4], and Martijn S. Luijsterburg[1*]

[1] Department of Human Genetics, Leiden University Medical Center, Leiden, The Netherlands
[2] Department of Genetics, Research Institute of Environmental Medicine, Nagoya University, Nagoya, Japan
[3] Department of Molecular Genetics, Center for Neurological Diseases and Cancer, Nagoya University Graduate School of Medicine, Nagoya, Japan
[4] Department of Human Genetics, Nagoya University Graduate School of Medicine, Nagoya, Japan

[#] Equal contribution

[*] **Corresponding author:**

Martijn S. Luijsterburg (m.luijsterburg@lumc.nl)

This PDF file includes:

Supplementary Figures 1–6

Supplementary Tables 1–5

Supplementary References

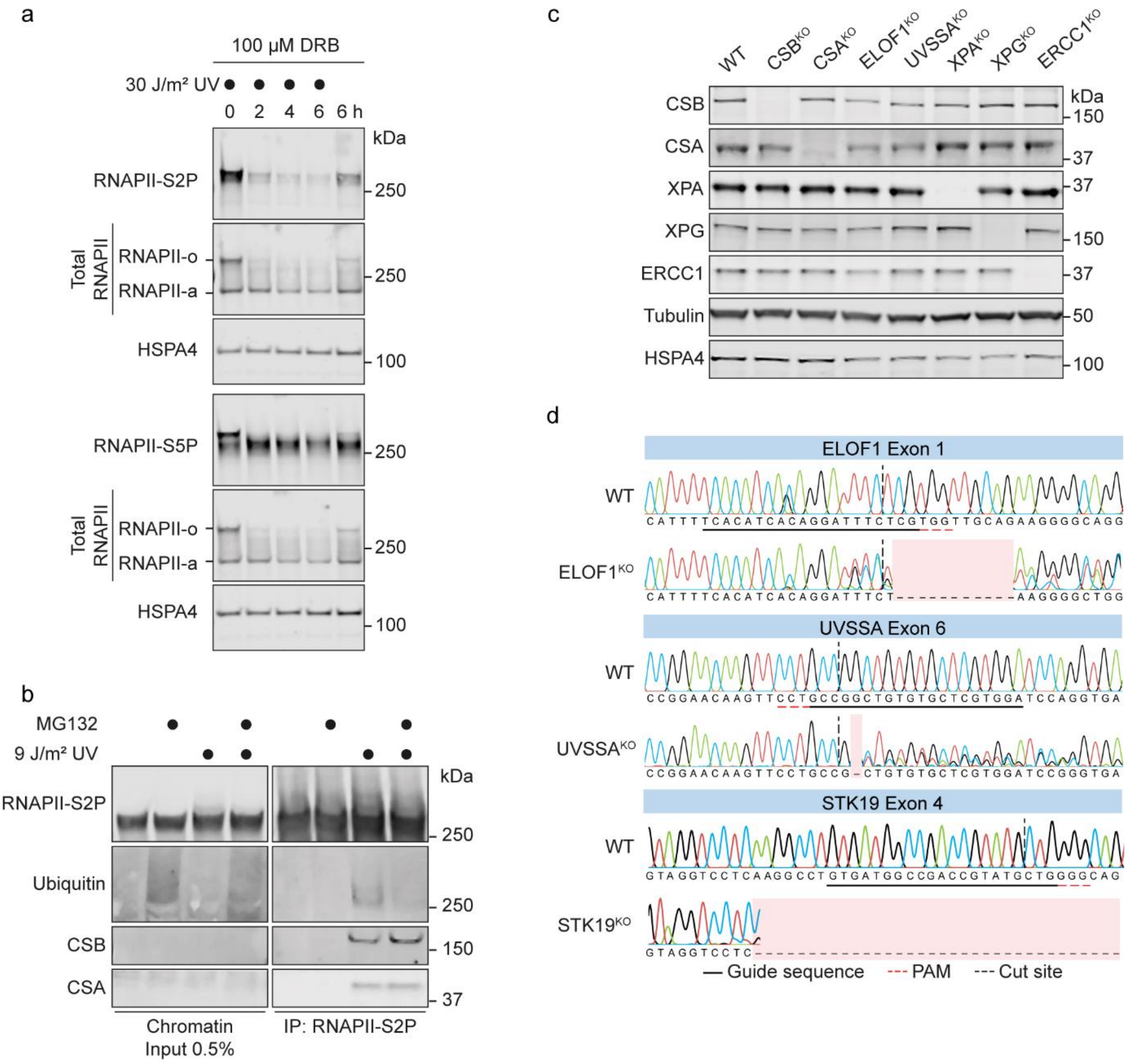

Supplementary Fig. 1 | Validation of the effect of DRB on the different RNAPII pools and the collection of TCR KO cell lines.

a. Detection of the different RNAPII pools by western blot from whole cell lysates of RPE1 WT cells at 0, 2, 4, and 6 h after global UV-C damage (30 J/m^2) and incubation with 100 µM DRB. An antibody against the N-terminus of RNAPII (Cell Signaling, 14958 (D8L4Y)) detects the total RNAPII pool. The higher-migrating band is the hyperphosphorylated/elongating RNAPII (RNAPIIo), which contains both S5P and S2P. The lower migrating band is the hypophosphorylated RNAPII (RNAPIIa), which is the promoter-bound form and contains mainly S5P. Top: Staining for RNAPII-S2P (Millipore, 04-1571 (3E10)) specifically reveals the elongating RNAPII pool that is rapidly degraded after UV-C exposure. Bottom: Staining for RNAPII-S5P (Millipore, 3E8 (04-1572-1)) reveals predominantly the promoter-proximal pause site-bound RNAPII. The S5P signal decreases gradually over time as transcription progresses and does not show a rapid degradation after UV-C exposure.

b. Detection of RNAPII-S2P ubiquitylation (Cell Signaling, 3936) by western blot after immunoprecipitation of RNAPII-S2P (right) from the chromatin fraction (left) of RPE1 WT cells. Cells were incubated with or without the proteasome MG132 (Merck, 474790; 20 µM) for 1 h

before 9 J/m^2 UV-C irradiation. Then, cells were incubated in the same media and collected 1 h after UV-C exposure.
c. Validation of the TCR knockouts by western blotting. The indicated antibodies were used to analyze whole cell lysates from parental and knockout RPE1 cell lines.
d. Validation of ELOF1^{KO}, UVSSA^{KO,} and STK19^{KO} cell lines by Sanger sequencing. Chromatographs of the genomic regions of the respective genes, expanding upstream and downstream of the cut site by the sgRNA.

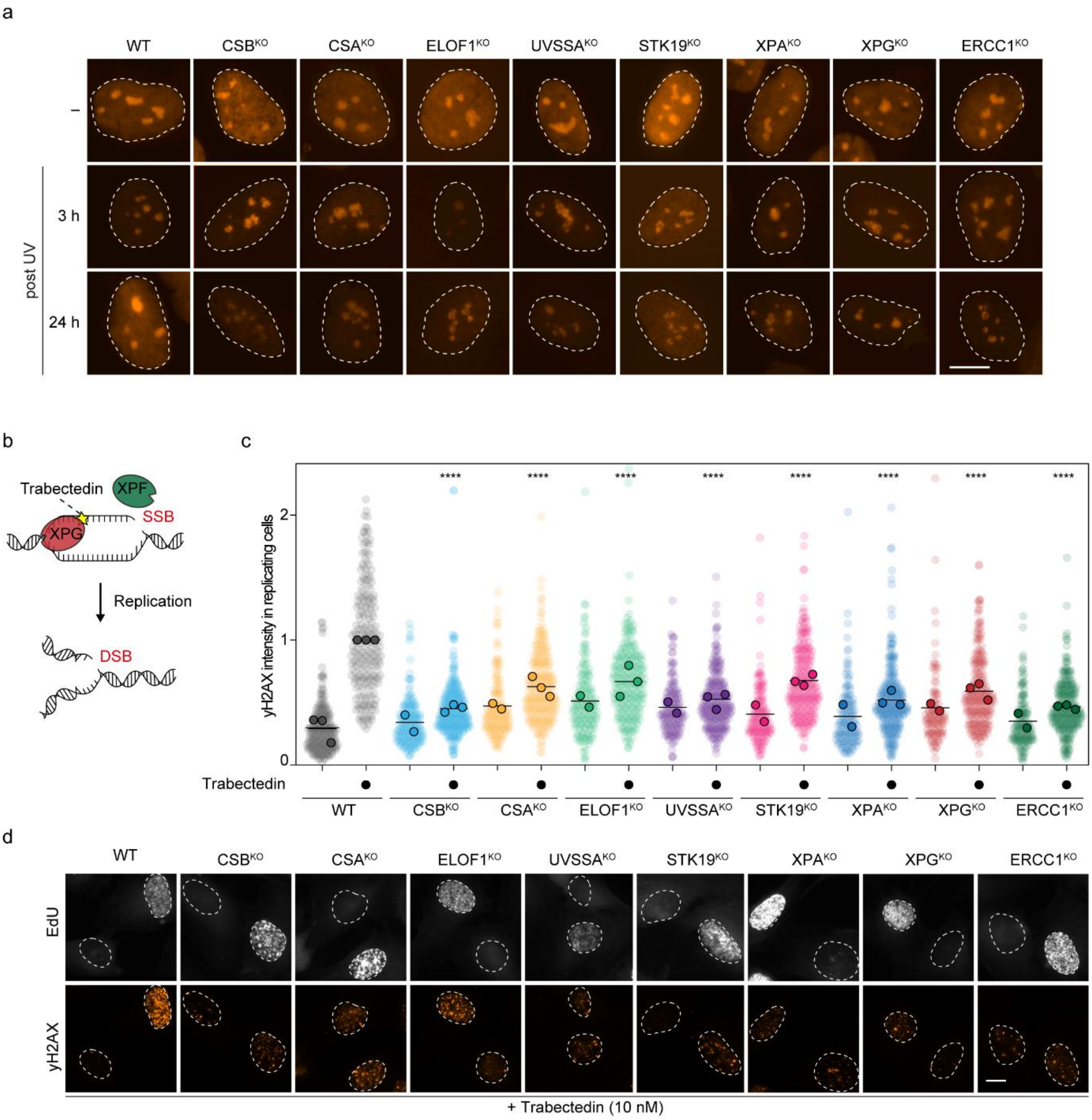

Supplementary Fig. 2 | TCR activity in isogenic TCR knockout cells

a. Representative images of the RRS in Figure 2a-b.

b. Model of TCR-dependent double-strand break formation in replicating cells resulting from trabectedin treatment.

c. Quantification of TCR-dependent γH2AX induction following trabectedin exposure in replicating cells labelled with 5-ethynyl-deoxyuridine (EdU) in RPE1 cells with the indicated genotype. The γH2AX levels were normalized to the average of the trabectedin-treated parental WT cells within each experiment. Each colored circle represents one cell. Each dark circle represents the mean of two technical replicates, with more than 50 cells collected per technical replicate. The black lines represent the mean of independent biological replicates.

d. Representative images of **c.** Scale bar, 10 µm.

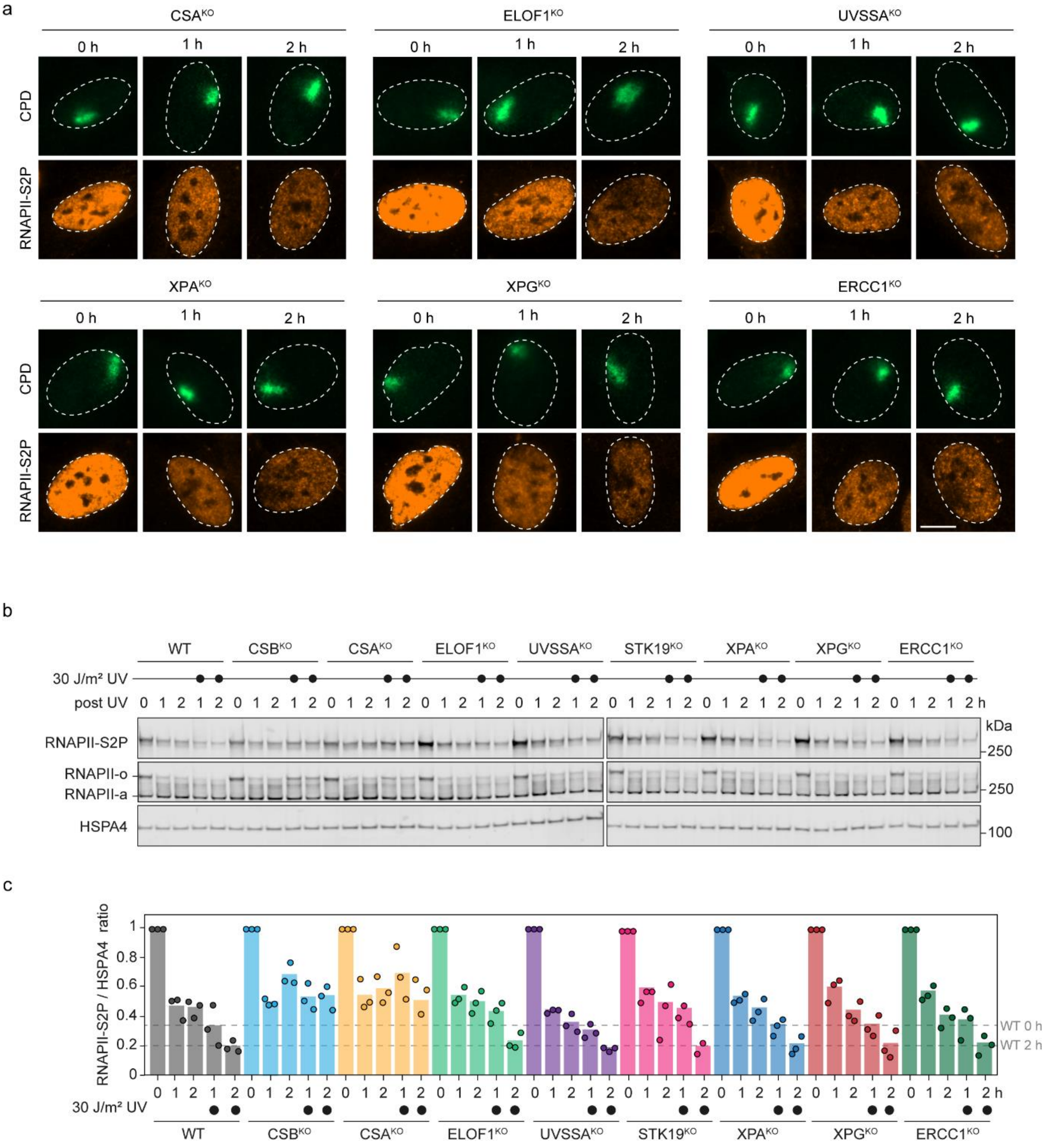

Supplementary Fig. 3 | Local clearance and global degradation of damage-stalled RNAPII in TCR knockout cells.

a. Representative images of the local DRB run-off immunofluorescence assay of RPE1 cells with the indicated genotypes that are quantified in Figure 2d. Scale bar, 10 µm.

b. Representative image of the global DRB run-off assay by western blotting of RPE1 WT and the indicated TCR knockout cells as described in Figure 1e.

c. Quantification of **b** as described in Figure 1f, used for calculating the RNAPII-S2 +UV / -UV ratios in Figure 2f.

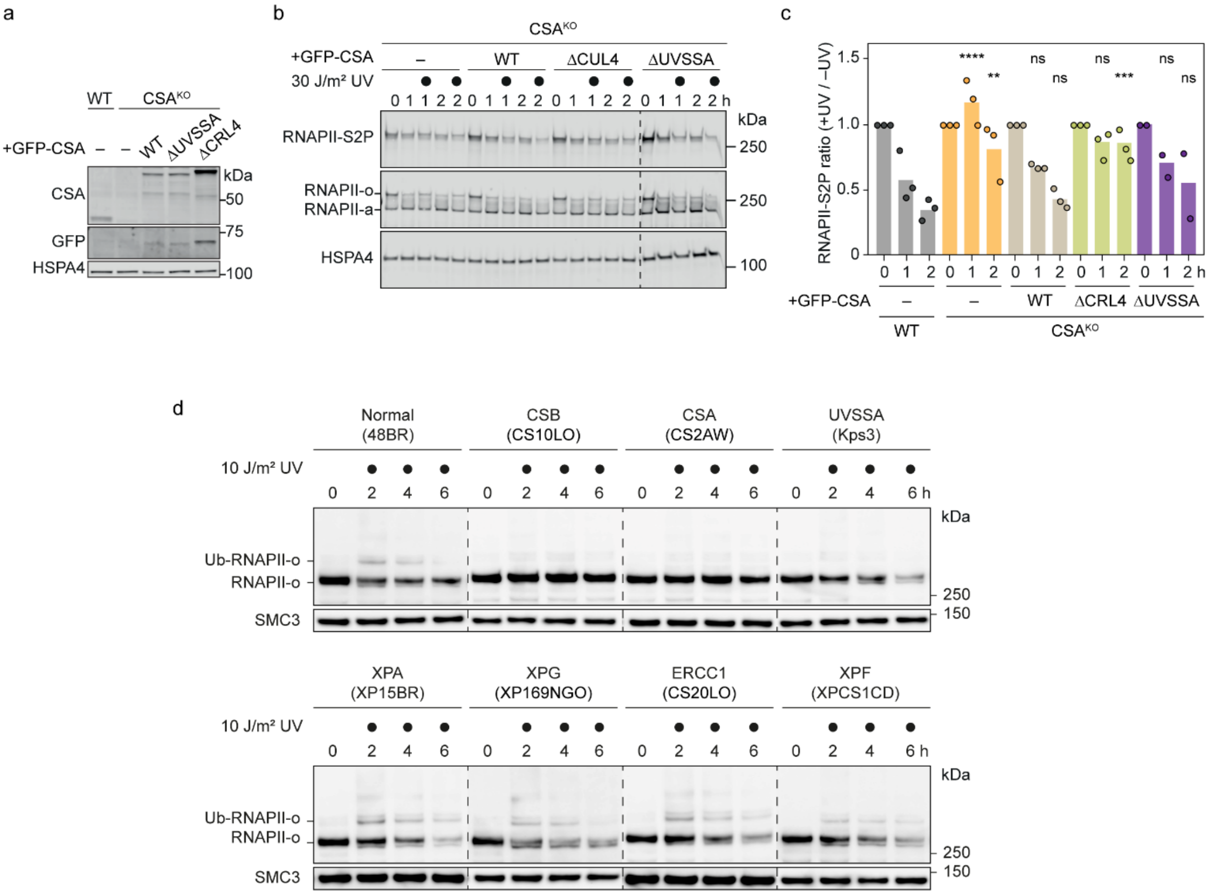

Supplementary Fig. 4 | Ubiquitylation of damage-stalled RNAPII is required for its clearance in RPE1 cells and primary fibroblasts.

a. Detection of CSA levels by western blot in whole cell lysates of RPE1 WT, CSA^{KO}, and CSA^{KO} stably expressing the indicated CSA proteins. HSPA4 was used as a loading control.

b. Representative image of the global DRB run-off assay by western blotting as described for Figure 1e, in RPE1 WT, CSA^{KO}, and CSA^{KO} stably expressing the indicated CSA constructs.

c. Quantification of **c** as described for Figure 1f-g.

d. Representative images of the RNAPII signal detected by western blot analysis from whole cell lysates of normal and the indicated TCR mutant primary fibroblasts at 0, 2, 4, and 6 h after global UV-C damage (10 J/m^2). Cells were treated with 100 μM cycloheximide 1 h before UV-C irradiation and incubated in the same media after UV-C exposure. An antibody against hyperphosphorylated C-terminal domain (CTD) Ser2/5 (BioLegend, 920203 (H5)) was used to detect the elongating RNAPIIo. The SMC3 signal was used as a loading control.

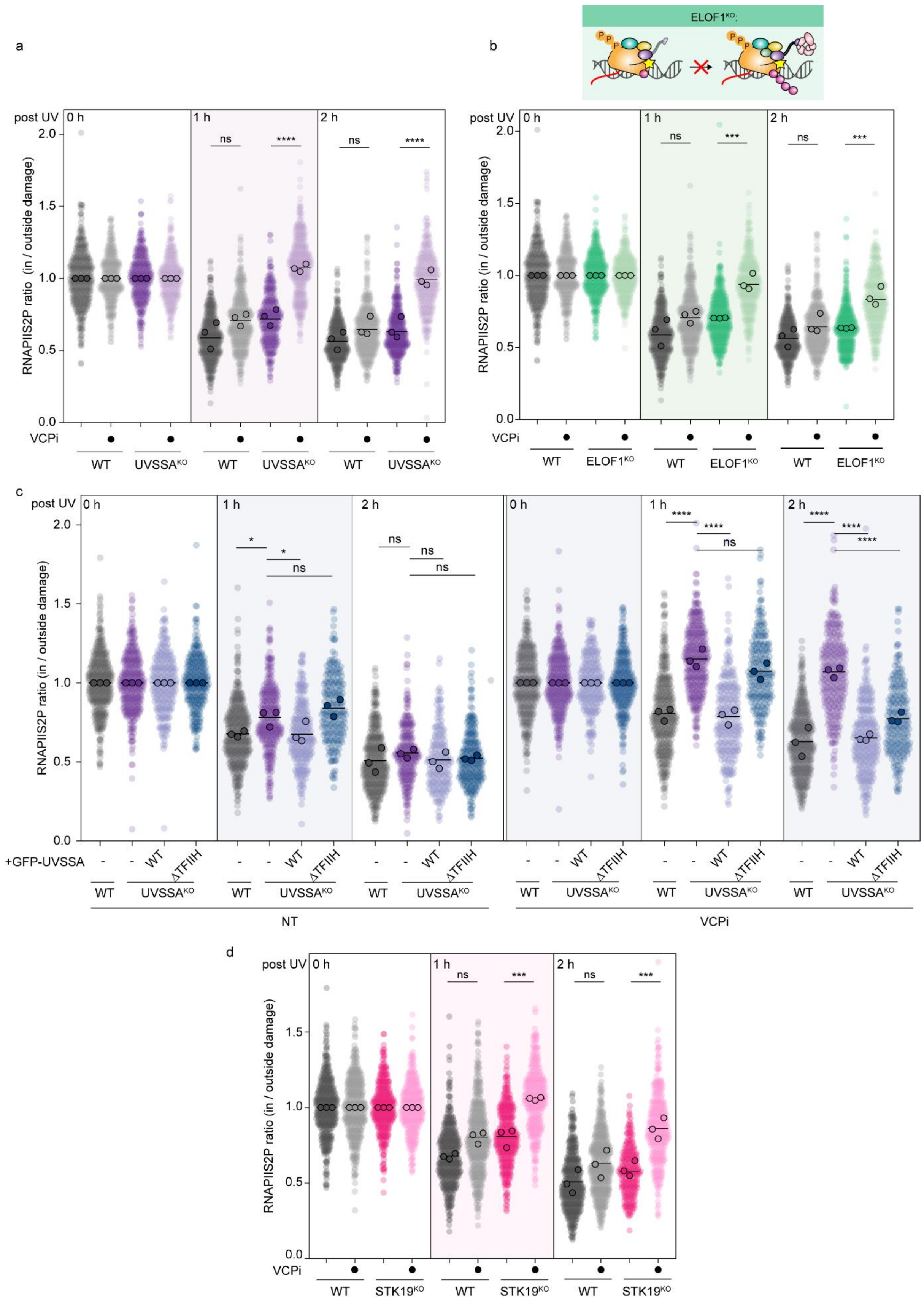
a
b
ELOF1KO:
post UV
0 h
1 h
2 h
ns
****
***
RNAPIIS2P ratio (in / outside damage)
VCPi
WT
UVSSAKO
ELOF1KO
c
*
+GFP-UVSSA
ΔTFIIH
NT
d
STK19KO

Supplementary Fig. 5 | Under-ubiquitylated RNAPII is cleared by the VCP segregase in the absence of TFIIH in the TCR complex

a. Quantification of the local DRB run-off immunofluorescence assay in Figure 4a, including the 2 h timepoint after UV-C treatment.

b. Top: Cartoon depicting the effect of ELOF^{KO} on TCR complex assembly. Bottom: Quantification of the local DRB run-off immunofluorescence assay as described for Figure 1a-c for 0, 1, and 2 h after local UV-C treatment in RPE1 WT and ELOF1^{KO} with or without VCPi treatment as in Figure 4a.

c. Quantification of the local DRB run-off immunofluorescence assay in Figure 4e, including the 2 h timepoint after UV-C treatment.

d. Quantification of the local DRB run-off immunofluorescence assay in Figure 4f, including the 2 h timepoint after UV-C treatment.

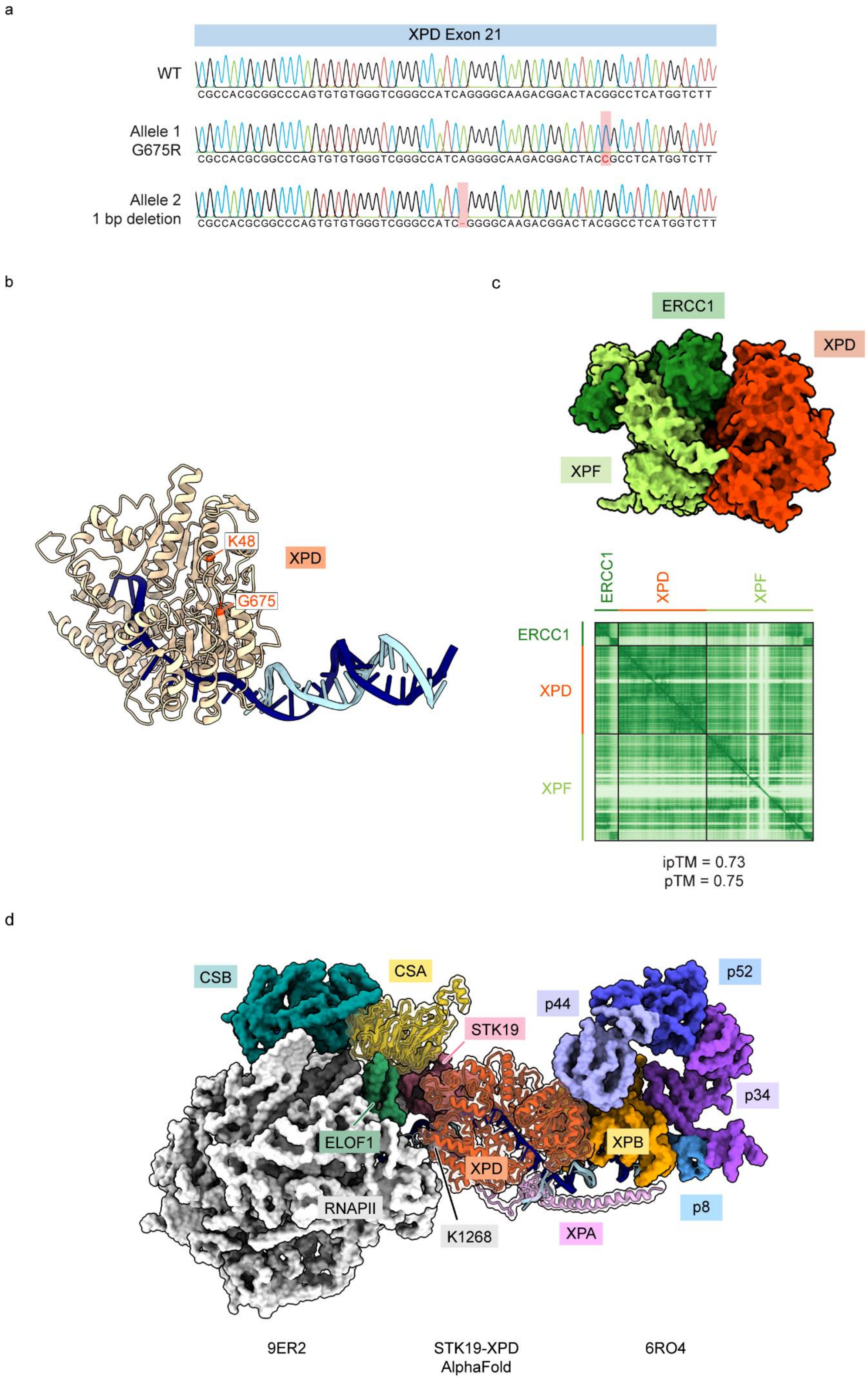
a
XPD Exon 21
WT
CGCCACGCGGCCCAGTGTGTGGGTCGGGCCATCAGGGGCAAGACGGACTACGGCCTCATGGTCTT
Allele 1
G675R
CGCCACGCGGCCCAGTGTGTGGGTCGGGCCATCAGGGGCAAGACGGACTACCGCCTCATGGTCTT
Allele 2
1 bp deletion
CGCCACGCGGCCCAGTGTGTGGGTCGGGCCATC-GGGGCAAGACGGACTACGGCCTCATGGTCTT
b
K48
G675
XPD
c
ERCC1
XPD
XPF
ERCC1
XPD
XPF
ERCC1
XPD
XPF
ipTM = 0.73
pTM = 0.75
d
CSB
CSA
STK19
p44
p52
p34
ELOF1
XPB
XPD
RNAPII
K1268
XPA
p8
9ER2
STK19-XPD
AlphaFold
6RO4

Supplementary Fig. 6 | XPD helicase in the TCR complex

a. Validation of the XPD helicase-dead fibroblast cell line by Sanger sequencing. Chromatographs of the genomic regions of the XPD mutation (G675R) on one allele and presumed KO on the other (-1 bp deletion located 17 bp upstream of the XPD mutation on the first allele).

b. XPD bound to DNA from Kokic et al. 2019 (PDB: 6RO4)[1]. Two amino acids are highlighted, G675 and K48, that are required for the helicase activity of XPD.

c. AlphaFold3 model of ERCC1-XPF (green) bound to XPD (red). The contact scores are shown below the model (ipTM = 0.73, pTM = 0.75).

d. Superimposition of the RNAPII-TCR-STK19 structure from Van den Heuvel et al. 2024 (PDB: 9ER2)[2] and TFIIH core complex from Kokic et al. 2019 (PDB:6RO4)[3] through the AlphaFold-predicted XPD-STK19 interaction[2]. Note that the XPD-STK19 interaction positions the TFIIH complex in front of RNAPII. The loop containing the K1268 ubiquitylation site in RPB1 was superimposed from Mevissen et al. 2024[4].

Supplementary Table 1: Cell lines

Cell lines	Source
RPE1-iCas9 (WT)	[5]
RPE1-iCas9 CSA-KO (3-1)	[5]
RPE1-iCas9 CSB-KO (1-15)	[5]
RPE1-iCas9 ELOF1-KO (2-16)	[5]
RPE1-iCas9 ERCC1-KO (16)	[6]
RPE1-iCas9 UVSSA-KO (3-9)	[5]
RPE1-iCas9 STK19-KO (7)	[2]
RPE1-iCas9 XPA-KO (1)	This study
RPE1-iCas9 XPG-KO (21)	[7]
48BR-hTERT (WT)	[8]
XP8BR-hTERT (XP-D/CS)	[8]
48BR (WT primary fibroblast)	[9]
CS10LO (CS-B primary fibroblast)	[10]
CS2AW (CS-A primary fibroblast)	[11]
Kps3 (UVSS-A primary fibroblast)	[12]
XP15BR (XP-A primary fibroblast)	[13]
XP169NGO (XP-G primary fibroblast)	This study
CS20LO (ERCC1 primary fibroblast)	[14]
XPCS1CD (XP-F primary fibroblast)	[14]

Supplementary Table 2: sgRNAs, crRNAs, siRNAs

RNA	Sequence	Gene_ID
sgCSB	CTCATCGGATCATTCTGTCT	ENSG00000225830
sgCSA	GGAGAGCAGAGTCAACACGG	ENSG00000049167
sgELOF1	CGAGAAATCCTGTGATGTGA	ENSG00000130165
sgUVSSA	TCCACGAGCACACAGCCGGC	ENSG00000163945
sgSTK19	GTGATGGCCGACCGTATGCTG	ENSG00000206342 (misannotated, should be 254 amino acids)
sgXPA	CCTGTGTCAATTATCTTTGGGGC	ENSG00000136936
sgXPG	GAAGGGAAGATCCTGGCTGT	ENSG00000134899
sgERCC1	GGTACCGCCCAGCTTCCTCGGGG	ENSG00000134899
siUVSSA	ON-TARGETplus Human UVSSA (L-024139-02-0005)	ENSG00000163945
crERCC2/XPD	AAGGAACAGGTGCTCACCTC	ENSG00000104884

Supplementary Table 3: Plasmids

Plasmids	Origin	Identifier
pX458	Addgene #48138	pML#006
pX458-(Cas9-2A-GFP)-sgCSB-1	[3]	pML#003
pX458-(Cas9-2A-GFP)-sgCSA-3	[3]	pML#219
pX458-(Cas9-2A-GFP)-sgELOF1-2	[3]	pML#186
pX458-(Cas9-2A-GFP)-sgUVSSA_3	[3]	pML#220
pX458-(Cas9-2A-GFP)-sgSTK19	[2]	pML#214
pLV-U6g-PPB-sgXPA_2	This study	sgML#002
pX458-(Cas9-2A-GFP)-sgXPG	[7]	pML#223
pLV-U6g-PPB-sgERCC1_1	[6]	sgML#049
pLenti_PGK_CSA^{WT}-GFP_Puro	[3]	pML#445
pLenti_PGK_CSA$^{\Delta CRL4}$-GFP_Puro	This study	pML#500
pLenti_PGK_CSA$^{\Delta UVSSA}$-GFP_Puro	[3]	pML#446
pLenti-pPGK-XPD^{WT}-GFP_Puro	This study	pML#553
pLenti-pPGK-XPD^{K48R}-GFP_Puro	This study	pML#620
pLenti-pPGK-XPD^{G675R}-GFP_Puro	This study	pML#619

Supplementary Table 4: Oligos

Gene	Sequence	Identifier
ELOF1	5-ATGTTGCCCAGGCTGGTATC-3	oML#320_sgELOF1-2_Seq_FW1
	5-TCCTCTGTGTCGCTACTGATTG-3	oML#321_sgELOF1-2_Seq_RV1
	5-GATCACAGGTGTGAGCCAC-3	oML#328_sgELOF1-2_Seq_FW2
	5-CACTTAGGTCAAGGGCGATC-3	oML#329_sgELOF1-2_Seq_RV2
UVSSA	5-ACCCAGAGGTACACAGAGATTG-3	oML#090_ sgUVSSA1-1_ FW
	5-GCTCTTAGAAGTGTCCCTGTG-3	oML#091_ sgUVSSA1-1_ RV
	5-ATCAGGAGGCTGAGGCGGCTG-3	oML#076_ sgUVSSA1-2_ FW
	5-AGGAGCCTACCCGGGAGCCGGG-3	oML#077_ sgUVSSA1-2_ RV
STK19	5-CTTCACTGAGGACTACAGGAC-3	oML#435_sgSTK19_Seq_Fw
	5-CCAGCTCCTACCATTCTCTATC-3	oML#436_sgSTK19_Seq_Rv
	5-CTCACATTGCCAAAAGACGGCA-3	oML#633_IRES_seq_Rv
	5-GGCCAATAGCGGCTGCTCAG-3	oML#634_PGK_seq_Fw
ERCC2/XPD	5-GTCCCCTACGTCTACACACAGAGCCG-3	oML#1475_XP8BRseq_Fw
	5-CGGTGGAAGGGCTGTGCCATC-3	oML#1476_XP8BRseq_Rv

Supplementary Table 5: Antibodies

Antibody	Host	Company (reference)	Use	Identifier
α-Tubulin	Mouse	Sigma, #T6199 (DM1A)	WB: 1:1000	aML#008
CPD	Mouse	Cosmo Bio, CAC-NM-DND-001	IF: 1:1000	aML#020
CSA/ERCC8	Rabbit	Abcam, #137033 (EPR9237)	WB: 1:1000	aML#028
CSB/ERCC6	Rabbit	Bethyl Laboratories, #A301-345A	WB: 1:600	aML#187
ERCC5/XPG	Rabbit	Bethyl Laboratories #A301-484A-2	WB: 1:1000	aML#138
ERCC1	Mouse	Santa Cruz, sc-17809	WB: 1:500	aML#066
GFP	Mouse	Roche, #11814460001	WB: 1:1000	aML#011
HDAC1	Rabbit	Abcam, ab19845	WB: 1:1000	aML#027
HSPA4	Rabbit	Novus Biologicals, NBP1-81696	WB: 1:1000	aML#114
Mouse Alexa 488	Goat	Thermo Fisher A-11029	IF: 1:1000	aML#013
Mouse Alexa 555	Goat	Thermo Fisher Scientific, A-21424	IF: 1:1000	aML#015
Mouse IgG (H+L) CF770	Goat	Biotium, VWR #20077	WB: 1:10000	aML#009
p62/GTF2H1	Mouse	Santa Cruz, #sc-48431 (G10)	WB: 1:1000	aML#099
p89/XPB/ERCC3	Mouse	Millipore, #MABE1123	WB: 1:1000	aML#101
phospho-H2A.X Ser139	Mouse	Merck, #05-636 (JBW301)	IF: 1:1000	aML#161
Rabbit Alexa 555	Goat	Thermo Fisher A-21429	IF: 1:1000	aML#014
Rabbit IgG (H+L) CF680	Goat	Biotum, VWR#: 20067	WB: 1:10000	aML#010
Rat IgG (H+L) CF770	Goat	Biotium, 20383	WB: 1:10000	aML#134
RNAPII CTD (S2/S5)	Mouse	BioLegend #920203 (H5)	WB: 1:1000	n.a.
RNAPII N-terminus	Rabbit	Cell Signaling, #14958 (D8L4Y)	WB: 1:1000	aML#252
RNAPII-S2P	Rabbit	Abcam, #ab5095	IF: 1:1000	aML#024
RNAPII-S2P	Rat	Millipore, #04-1571 (3E10)	WB: 1:1000	aML#120
RNAPII-S5P	Rat	Millipore, #3E8 (04-1572-1)	WB: 1:1000	aML#133
SMC3	Rabbit	Bethyl laboratories #A300-060A	WB: 1:4000	n.a.
Ubiquitin	Mouse	Cell Signaling, #3936	WB: 1:1000	aML#190
XPA	Rabbit	gift of Rick Wood (CJ1)	WB: 1:5000	aML#079
XPD/ERCC2	Mouse	Abcam, ab54676	WB: 1:1000	aML#029

Supplementary References

1. Kokic, G. et al. Structural basis of TFIIH activation for nucleotide excision repair. *Nat Commun* **10**, 2885 (2019).
2. van den Heuvel, D. et al. STK19 facilitates the clearance of lesion-stalled RNAPII during transcription-coupled DNA repair. *Cell* (2024).
3. Kokic, G. et al. Structural basis for RNA polymerase II ubiquitylation and inactivation in transcription-coupled repair. *Nat Struct Mol Biol* **31**, 536–547 (2024).
4. Mevissen, T.E.T., Kummecke, M., Schmid, E.W., Farnung, L. & Walter, J.C. STK19 positions TFIIH for cell-free transcription-coupled DNA repair. *Cell* (2024).
5. van der Weegen, Y. et al. ELOF1 is a transcription-coupled DNA repair factor that directs RNA polymerase II ubiquitylation. *Nat Cell Biol* **23**, 595–607 (2021).
6. Apelt, K. et al. ERCC1 mutations impede DNA damage repair and cause liver and kidney dysfunction in patients. *J Exp Med* **218**(2021).
7. van der Meer, P.J., Van Den Heuvel, D. & Luijsterburg, M.S. Unscheduled DNA Synthesis at Sites of Local UV-induced DNA Damage to Quantify Global Genome Nucleotide Excision Repair Activity in Human Cells. *Bio Protoc* **13**(2023).
8. Broughton, B.C. et al. Molecular and cellular analysis of the DNA repair defect in a patient in xeroderma pigmentosum complementation group D who has the clinical features of xeroderma pigmentosum and Cockayne syndrome. *Am J Hum Genet* **56**, 167–74 (1995).
9. Arlett, C.F., Green, M.H., Priestley, A., Harcourt, S.A. & Mayne, L.V. Comparative human cellular radiosensitivity: I. The effect of SV40 transformation and immortalisation on the gamma-irradiation survival of skin derived fibroblasts from normal individuals and from ataxia-telangiectasia patients and heterozygotes. *Int J Radiat Biol* **54**, 911–28 (1988).
10. Senju, C. et al. Aicardi-Goutieres syndrome with SAMHD1 deficiency can be diagnosed by unscheduled DNA synthesis test. *Front Pediatr* **10**, 1048002 (2022).
11. Ren, Y. et al. Three novel mutations responsible for Cockayne syndrome group A. *Genes Genet Syst* **78**, 93–102 (2003).
12. Nakazawa, Y. et al. Mutations in UVSSA cause UV-sensitive syndrome and impair RNA polymerase IIo processing in transcription-coupled nucleotide-excision repair. *Nat Genet* **44**, 586–92 (2012).
13. Fassihi, H. et al. Deep phenotyping of 89 xeroderma pigmentosum patients reveals unexpected heterogeneity dependent on the precise molecular defect. *Proc Natl Acad Sci U S A* **113**, E1236–45 (2016).
14. Kashiyama, K. et al. Malfunction of nuclease ERCC1-XPF results in diverse clinical manifestations and causes Cockayne syndrome, xeroderma pigmentosum, and Fanconi anemia. *Am J Hum Genet* **92**, 807–19 (2013).
