# Supplementary material for: Hierarchical mechanisms control the clearance of DNA lesion–stalled RNA polymerase II
Source: Nat Commun. 2026 Jan 19;17:1647. doi: 10.1038/s41467-026-68413-4 (PMC12904859; doi:10.1038/s41467-026-68413-4)
Supplement: Supplementary file 2 — Reporting Summary [file 41467_2026_68413_MOESM2_ESM.pdf]

nature portfolio

Corresponding author(s): Martijn S. Luijsterburg
Last updated by author(s): Sep 29, 2025

Reporting Summary

Nature Portfolio wishes to improve the reproducibility of the work that we publish. This form provides structure for consistency and transparency in reporting. For further information on Nature Portfolio policies, see our Editorial Policies and the Editorial Policy Checklist.

Statistics

For all statistical analyses, confirm that the following items are present in the figure legend, table legend, main text, or Methods section.

n/a	Confirmed	
☐	☒	The exact sample size (*n*) for each experimental group/condition, given as a discrete number and unit of measurement
☐	☒	A statement on whether measurements were taken from distinct samples or whether the same sample was measured repeatedly
☐	☒	The statistical test(s) used AND whether they are one- or two-sided<br>*Only common tests should be described solely by name; describe more complex techniques in the Methods section.*
☒	☐	A description of all covariates tested
☐	☒	A description of any assumptions or corrections, such as tests of normality and adjustment for multiple comparisons
☐	☒	A full description of the statistical parameters including central tendency (e.g. means) or other basic estimates (e.g. regression coefficient) AND variation (e.g. standard deviation) or associated estimates of uncertainty (e.g. confidence intervals)
☐	☒	For null hypothesis testing, the test statistic (e.g. *F*, *t*, *r*) with confidence intervals, effect sizes, degrees of freedom and *P* value noted<br>*Give P values as exact values whenever suitable.*
☒	☐	For Bayesian analysis, information on the choice of priors and Markov chain Monte Carlo settings
☒	☐	For hierarchical and complex designs, identification of the appropriate level for tests and full reporting of outcomes
☒	☐	Estimates of effect sizes (e.g. Cohen's *d*, Pearson's *r*), indicating how they were calculated

Our web collection on statistics for biologists contains articles on many of the points above.

Software and code

Policy information about availability of computer code

Data collection	Microscopy images were acquired using a Zeiss AxioImager M2 widefield fluorescence microscope and ZEN 2012 software (blue edition, version 1.1.0.0). Western blot images were acquired using a Odyssey CLx with Image studio lite software (v5.2) or for western blot with ECL a Amersham Imager 680.
Data analysis	Microscopy images were analyzed in Image J (1.48v). Graphs were plotted and analyzed using Plots of Data. Western blot images were analyzed in Image Studio Lite (v5.0.21). Statistical analyses were performed in GraphPad Prism version 10.2.3 (403).

For manuscripts utilizing custom algorithms or software that are central to the research but not yet described in published literature, software must be made available to editors and reviewers. We strongly encourage code deposition in a community repository (e.g. GitHub). See the Nature Portfolio guidelines for submitting code & software for further information.

Data

Policy information about availability of data

All manuscripts must include a data availability statement. This statement should provide the following information, where applicable:

- Accession codes, unique identifiers, or web links for publicly available datasets
- A description of any restrictions on data availability
- For clinical datasets or third party data, please ensure that the statement adheres to our policy

This published article (and its supplementary information files) includes all data generated or analyzed during this study.

Research involving human participants, their data, or biological material

Policy information about studies with human participants or human data. See also policy information about sex, gender (identity/presentation), and sexual orientation and race, ethnicity and racism.

Reporting on sex and gender	*Use the terms sex (biological attribute) and gender (shaped by social and cultural circumstances) carefully in order to avoid confusing both terms. Indicate if findings apply to only one sex or gender; describe whether sex and gender were considered in study design; whether sex and/or gender was determined based on self-reporting or assigned and methods used. Provide in the source data disaggregated sex and gender data, where this information has been collected, and if consent has been obtained for sharing of individual-level data; provide overall numbers in this Reporting Summary. Please state if this information has not been collected. Report sex- and gender-based analyses where performed, justify reasons for lack of sex- and gender-based analysis.*
Reporting on race, ethnicity, or other socially relevant groupings	*Please specify the socially constructed or socially relevant categorization variable(s) used in your manuscript and explain why they were used. Please note that such variables should not be used as proxies for other socially constructed/relevant variables (for example, race or ethnicity should not be used as a proxy for socioeconomic status). Provide clear definitions of the relevant terms used, how they were provided (by the participants/respondents, the researchers, or third parties), and the method(s) used to classify people into the different categories (e.g. self-report, census or administrative data, social media data, etc.) Please provide details about how you controlled for confounding variables in your analyses.*
Population characteristics	*Describe the covariate-relevant population characteristics of the human research participants (e.g. age, genotypic information, past and current diagnosis and treatment categories). If you filled out the behavioural & social sciences study design questions and have nothing to add here, write "See above."*
Recruitment	*Describe how participants were recruited. Outline any potential self-selection bias or other biases that may be present and how these are likely to impact results.*
Ethics oversight	*Identify the organization(s) that approved the study protocol.*

Note that full information on the approval of the study protocol must also be provided in the manuscript.

Field-specific reporting

Please select the one below that is the best fit for your research. If you are not sure, read the appropriate sections before making your selection.

☒ Life sciences ☐ Behavioural & social sciences ☐ Ecological, evolutionary & environmental sciences

For a reference copy of the document with all sections, see nature.com/documents/nr-reporting-summary-flat.pdf

Life sciences study design

All studies must disclose on these points even when the disclosure is negative.

Sample size	No statistical method was used to predetermine sample size. Sample sizes were chosen for the different experimental approaches based on the technical difficulty and throughput of the individual assays, the chosen sample sizes are consistent with previous publications.
Data exclusions	No data was excluded.
Replication	All replication attempts were successful. The number of replicate experiments are indicated in the figure legends of the manuscript and three replicates were performed for each individual approach.
Randomization	There was no allocation of test subjects for any experiments, thus randomization was not applicable to our study
Blinding	Data analyses were performed by unbiased software programs/algorithms blinding was therefore not applicable to our study

Reporting for specific materials, systems and methods

We require information from authors about some types of materials, experimental systems and methods used in many studies. Here, indicate whether each material, system or method listed is relevant to your study. If you are not sure if a list item applies to your research, read the appropriate section before selecting a response.

Materials & experimental systems

n/a	Involved in the study
☐	☒ Antibodies
☐	☒ Eukaryotic cell lines
☒	☐ Palaeontology and archaeology
☒	☐ Animals and other organisms
☒	☐ Clinical data
☒	☐ Dual use research of concern
☒	☐ Plants

Methods

n/a	Involved in the study
☒	☐ ChIP-seq
☒	☐ Flow cytometry
☒	☐ MRI-based neuroimaging

Antibodies

Antibodies used

a-Tubulin Mouse Sigma, #T6199 (DM1A) WB: 1:1000 aML#008
CPD Mouse Cosmo Bio, CAC-NM-DND-001 IF: 1:1000 aML#020
CSA/ERCC8 Rabbit Abcam, #137033 (EPR9237) WB: 1:1000 aML#028
CSB/ERCC6 Rabbit Bethyl Laboratories, #A301-345A WB: 1:600 aML#187
ERCC5/XPG Rabbit Bethyl Laboratories #A301-484A-2 WB: 1:1000 aML#138
ERCC1 Mouse Santa Cruz, sc-17809 WB: 1:500 aML#066
GFP Mouse Roche, #11814460001 WB: 1:1000 aML#011
HDAC1 Rabbit Abcam, ab19845 WB: 1:1000 aML#027
HSPA4 Rabbit Novus Biologicals, NBP1-81696 WB: 1:1000 aML#114
Mouse Alexa 488 Goat Thermo Fisher A-11029 IF: 1:1000 aML#013
Mouse Alexa 555 Goat Thermo Fisher Scientific, A-21424 IF: 1:1000 aML#015
Mouse IgG (H+L) CF770 Goat Biotium, VWR #20077 WB: 1:10000 aML#009
p62/GTF2H1 Mouse Santa Cruz, #sc-48431 (G10) WB: 1:1000 aML#099
p89/XPB/ERCC3 Mouse Millipore, #MABE1123 WB: 1:1000 aML#101
phospho-H2A.X Ser139 Mouse Merck, #05-636 (JBW301) IF: 1:1000 aML#161
Rabbit Alexa 555 Goat Thermo Fisher A-21429 IF: 1:1000 aML#014
Rabbit IgG (H+L) CF680 Goat Biotum, VWR#: 20067 WB: 1:10000 aML#010
Rat IgG (H+L) CF770 Goat Biotium, 20383 WB: 1:10000 aML#134
RNAPII CTD (S2/S5) Mouse BioLegend #920203 (H5) WB: 1:1000 n.a.
RNAPII N-terminus Rabbit Cell Signaling, #14958 (D8L4Y) WB: 1:1000 aML#252
RNAPII-S2P Rabbit Abcam, #ab5095 IF: 1:1000 aML#024
RNAPII-S2P Rat Millipore, #04-1571 (3E10) WB: 1:1000 aML#120
RNAPII-S5P Rat Millipore, #3E8 (04-1572-1) WB: 1:1000 aML#133
SMC3 Rabbit Bethyl laboratories #A300-060A WB: 1:4000 n.a.
Ubiquitin Mouse Cell Signaling, #3936 WB: 1:1000 aML#190
XPA Rabbit gift of Rick Wood (CJ1) WB: 1:5000 aML#079
XPD/ERCC2 Mouse Abcam, ab54676 WB: 1:1000 aML#029

Validation

The following antibodies were validated in knockout cell lines:
CSA/ERCC8 Abcam, #137033 (EPR9237)
CSB/ERCC6 Bethyl Laboratories, #A301-345A
XPA gift of Rick Wood (CJ1)
ERCC5/XPG Bethyl Laboratories #A301-484A-2
ERCC1 Santa Cruz, sc-17809

The following antibodies are commonly used as RNA polymerase II specific antibodies:
RNAPII-S2 Abcam, #ab5095
RNAPII N-terminus Cell Signaling, #14958 (D8L4Y)
RNAPII-S2 Millipore, #04-1571 (3E10)
RNAPII CTD (S2/S5)

The following antibodies are commonly used as a loading control:
a-Tubulin Sigma, #T6199 (DM1A)
HSPA4 Novus Biologicals, NBP1-81696
SPT5 Santa Cruz, sc-133217g
SMC3 Bethyl laboratories #A300-060A

UV-specific staining that disappears with expected kinetics and persists in XPC-KO cells:
CPD Cosmo Bio, CAC-NM-DND-001

Eukaryotic cell lines

Policy information about cell lines and Sex and Gender in Research

Cell line source(s)

RPE1-iCas9 (WT) (van der Weegen et al., 2021)
RPE1-iCas9 CSA-KO (3-1) (van der Weegen et al., 2021)
RPE1-iCas9 CSB-KO (1-15) (van der Weegen et al., 2021)

RPE1-iCas9 ELOF1-KO (2-16) (van der Weegen et al., 2021)
RPE1-iCas9 UVSSA-KO (3-9) (van der Weegen et al., 2021)
RPE1-iCas9 XPA-KO (1) This study
RPE1-iCas9 XPG-KO (21) (van der Meer et al., 2023)
RPE1-iCas9 ERCC1-KO (16) (Apelt et al., 2021)
48BR (WT primary fibroblast) (Arlett et al., 1988)
CS10LO (CS-B primary fibroblast) (Senju et al., 2022)
CS2AW (CS-A primary fibroblast) (Ren et al., 2003)
Kps3 (UVSS-A primary fibroblast) (Nakazawa et al., 2012)
XP15BR (XP-A primary fibroblast) (Fassihi et al., 2016)
XP169NGO (XP-G primary fibroblast) This study
CS20LO (ERCC1 primary fibroblast) (Kashiyama et al., 2013)
XPCS1CD (XP-F primary fibroblast) (Kashiyama et al., 2013)

Authentication
All knockout cells were validated by western blot analysis and DNA sequencing

Mycoplasma contamination
All cell lines were routinely tested for mycoplasma and were nested negative

Commonly misidentified lines
(See ICLAC register)
No commonly misidentified cell lines were used in this study

Plants

Seed stocks
Report on the source of all seed stocks or other plant material used. If applicable, state the seed stock centre and catalogue number. If plant specimens were collected from the field, describe the collection location, date and sampling procedures.

Novel plant genotypes
Describe the methods by which all novel plant genotypes were produced. This includes those generated by transgenic approaches, gene editing, chemical/radiation-based mutagenesis and hybridization. For transgenic lines, describe the transformation method, the number of independent lines analyzed and the generation upon which experiments were performed. For gene-edited lines, describe the editor used, the endogenous sequence targeted for editing, the targeting guide RNA sequence (if applicable) and how the editor was applied.

Authentication
Describe any authentication procedures for each seed stock used or novel genotype generated. Describe any experiments used to assess the effect of a mutation and, where applicable, how potential secondary effects (e.g. second site T-DNA insertions, mosiacism, off-target gene editing) were examined.
